# 2020 - State of our *JCMR*

**DOI:** 10.1186/s12968-020-00704-1

**Published:** 2021-01-12

**Authors:** Warren J. Manning

**Affiliations:** Beth Israel Deaconess Medical Center, 330 Brookline Avenue, Boston, MA 02215 USA

**Keywords:** Cardiovascular magnetic resonance, Review, Editorial process, Imaging

## Abstract

There were 79 articles published in the *Journal of Cardiovascular Magnetic Resonance* (*JCMR*) in 2019, including 65 original research papers, 2 reviews, 8 technical notes, 1 Society for Cardiovascular Magnetic Resonacne (SCMR) guideline, and 3 corrections. The volume was down slightly from 2018 (n = 89) with a corresponding 5.5% increase in manuscript submissions from 345 to 366. This led to a slight decrease in the acceptance rate from 25 to 22%. The quality of the submissions continues to be high. The 2019 JCMR Impact Factor (which is published in June 2020) increased from 5.07 to 5.36. The 2020 impact factor means that on average, each *JCMR* published in 2017 and 2018 was cited 5.36 times in 2019. Our 5 year impact factor was 5.2. We are now finishing the 13th year of *JCMR* as an open-access publication with BMC. As outlined in this report, the Open-Access system has dramatically increased the reading and citation of *JCMR* publications. I hope that our authors will continue to send their very best, high quality manuscripts for *JCMR* consideration and that our readers will continue to look to *JCMR* for the very best/state-of-the-art publications in our field. It takes a village to run a journal. *JCMR* is blessed to have very dedicated Associate Editors, Guest Editors, and Reviewers. I thank each of them for their efforts to ensure that the review process occurs in a timely and responsible manner. These efforts have allowed the *JCMR* to continue as the premier journal of our field. My role, and the entire process would not be possible without the dedication and efforts of our managing editor, Diana Gethers (who will leaving the journal in the coming months) and our assistant managing editor, Jennifer Rodriguez, who has agreed to increase her reponsibilities. Finally, I thank you for entrusting me with the editorship of the *JCMR.* As I begin my 5^th^ year as your editor-in-chief, please know that I fully recognize we are not perfect in our review process. We try our best to objectively assess every submission in a timely manner, but sometimes don't get it “right.” The editorial process is a tremendously fulfilling experience for me. The opportunity to review manuscripts that reflect the best in our field remains a great joy and a highlight of my week!

## Background

In accordance with Open-Access publishing guidelines of our publisher, BMC, the *Journal of Cardiovascular Magnetic Resonance (JCMR)* articles are published on-line in a continuus fashion in chronologic order of acceptance, with no collating of the articles into sections or special thematic issues. For this reason, the Open-Access Editors had felt that it was useful for the *JCMR* audience to annually summarize the publications into broad areas of interest or themes, so that readers could view areas of interest in a single article in relation to each other and contemporaneous *JCMR* publications. Though I feel this information is quite valuable for our readership, this year I have chosen not to include this section so as to decrease our self-citation rate. I will instead, focus as before on conveying information regarding the editorial process and as a “State of our *JCMR*” summary.

**Fig. 1:**
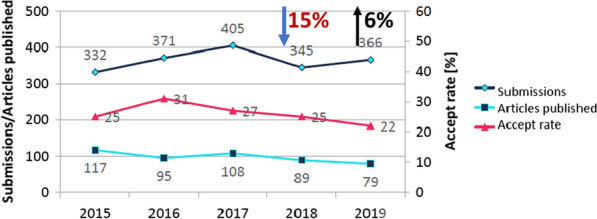
2015–2019 *JCMR* annual submissions, acceptances and acceptance rates. There was a 15% decrease in submissions in 2018, the year the article processing charge (APC) was changed to $500 for SCMR members. The submission volume increased by 6% in 2019

The *JCMR* is the official publication of the Society for Cardiovascular Magnetic Resonance (SCMR). There were 79 articles published in JCMR in 2019, including 65 original research papers, 2 reviews, 8 technical notes, 1 Society for Cardiovascular Magnetic Resonance (SCMR) guideline, and 3 corrections. The volume was down slightly from 2018 (n = 89) with a corresponding 6% increase in manuscript submissions from 345 to 366 (Fig. 1). As a result, there was a slight decrease in the acceptance rate from 25 to 22% though this is somewhat skewed in that manuscripts published in the first half of 2019 were likely submitted in 2018.

**Fig. 2:**
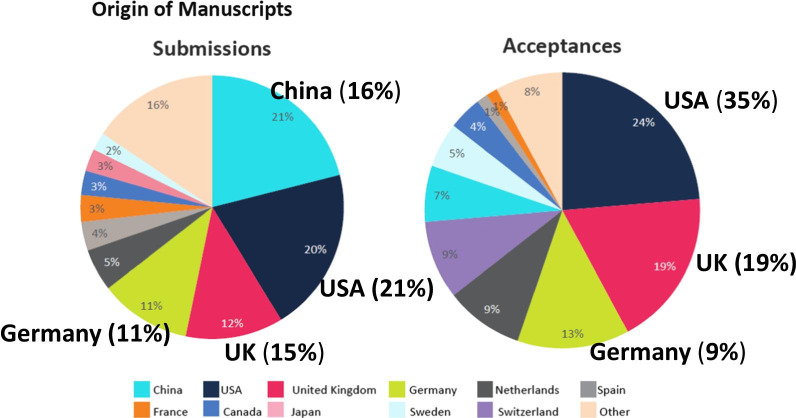
Country source of *JCMR* 2019 manuscript submissions and acceptances

For the first time, in 2019, the largest country source of annual submissions was China (n = 77). This was followed closely by the United States (n = 75) and then by the United Kingdom (n = 44) and Germany (n = 42) (Fig. 2). The top four publication countries were the United States (n = 18), United Kingdom (n = 14), Germany (n = 10), Switzerland (n = 7) and the Netherlands (n = 7) (Fig. 2). SCMR members continue to receive a substantial (80% discount) in the $2500 article processing charge (APC). Reduced APC fees are also available to those from BMC membership institutions, submitting authors from lower income countries, and for those who request a waiver due to financial hardship.

Though not the only journal success metric and not a consideration in our review process, the Impact Factor is nonetheless a well-known metric with which many are familiar and often considered for by both authors and readers. I am pleased to report that the 2019 *JCMR* Impact Factor (which was published in June 2020 and is based on manuscripts published in 2017 and 2018 that were cited in 2019) inceased to 5.36 (vs. 5.07 for 2018). The 2019 impact factor means that the *JCMR* papers that were published in 2017 and 2018 were cited on average 5.36 times in 2019. This puts *JCMR* well positioned in the top quartile of journals in the broad categories of “Cardiac and Cardiovascular Systems (23/138)” and “Radiology, Nuclear Medicine and Medical Imaging (13/133).” I fully anticipate that our 2020 Impact Factor will *decline* due to the change in the format of this manuscript and my decision to not include a thematic organization and overview of each of the prior two year’s publications. Most importantly, the open-access format allows for much greater visibility for our authors with *JCMR* annual digital accesses continuing to exceed 1,000,000—a threshold/visibility simply not achievable with a subspecialty journal as a subscription print publication. Open-access “leveled the playing field” so that an electronic search allows *JCMR* manuscripts to rise to awareness and to be downloaded without cost.

## *JCMR* Leadership

Dr. Gerald Pohost from the University of Alabama at Birmingham and University of Southern California, Los Angeles, California, USA was the *JCMR* inaugural editor-in-chief. In 2006, Dr. Pohost was succeeded by Professor Dudley Pennell of the Royal Brompton Hospital, London, England. Since December 2016, the *JCMR* editorial office has been located at the Beth Israel Deaconess Medical Center, Boston, Massachusetts, USA under the leadership of its third editor-in-chief, Dr. Warren J. Manning.

## 2020 *JCMR* Team and Personnel changes

**Fig. 3:**
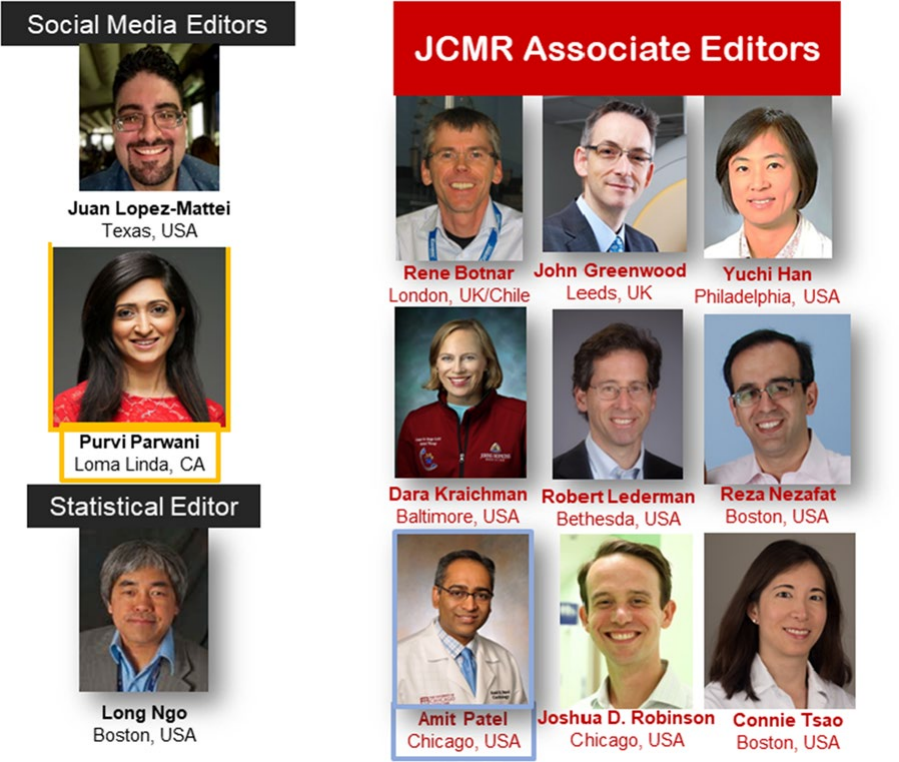
New members of the *JCMR* Associate Editorial board include Drs. Amit Patel (USA) and Connie Tsao (USA). Dr. Purvi Parwani joined as our new Social Media/Twitter Co-editors. Continuing members include Drs. Rene Botnar, John Greenwood, Yuchi Han, Dara Kraichman, Robert Lederman, Reza Nezafat, and Joshua Robinson

The current *JCMR* Associate Editors reflect the international and diverse spectrum of the SCMR. This past year, Dr. Tim Leiner stepped down from his associate editor *JCMR* position to undertake his term as the president of the International Society of Magnetic Resonance in Medicine (ISMRM). We have missed Tim, though he has graciously served as a Guest Editor for several manuscripts this year. This year we were fortunate to attract Drs. Amit Patel (USA) and Connie Tsao (USA) to the associate editorial team (Fig. 3); with a focus on cardiomyopathies and epidemiologic studies, respectively. Our other Associate Editors include Drs. Rene Botnar (UK/Chile), John Greenwood (UK), Yuchi Han (USA), Dara Kraichman (USA), Robert Lederman (USA), and Reza Nezafat (USA). In addition, Dr. Long Ngo (USA) serves as our statistical editor. Drs. Juan Lopez-Mattei (USA) was joined by Dr. Purvi Parwani (USA) as our Social Media/Twitter editors. Jennifer Rodriguez joined our managing editorial team mid year and and our managing editor, Diana Gethers (jcmroffice@scmr.org) has announced she will be leaving the *Journal* in the coming months. All correspondence to the *JCMR* managing office should continue to be sent to jcmroffice@scmr.org. The use of this “generic” email address allows for seamless communication during these transitions.

## Manuscript review process, omissions, and suggestions

I reviewed the manuscript submission process in my report last year [1] and will expand on this.

All manuscripts are submitted and processed through the http://www.jcmr-online.org website. I encourage all authors to closely follow the guidelines so as not to delay the review process. By far, the most common omission is to include the names and contact information for *at least two suggested reviewers* in their cover letter. I also ask authors to use *JCMR* preferred abbreviations (https://jcmr-online.biomedcentral.com/submission-guidelines/preparing-your-manuscript/abbreviations) and to use the terms “CMR” and cardiovascular magnetic resonance rather than cardiac magnetic resonance. While the abbreviation issue does not delay the review, it adds additional burden to the the prepublication editing process.

I also encourage all authors to carefully consider the number of significant digits and reported p values in their manuscripts. For example, when reporting native T1, values and standard deviation to the nearest ms should be reported and not to the X.X or X.XX ms which have no real substance. Similarly, when reporting p values for the sample sizes of most *JCMR* publications, a value of < 0.001 is a reasonable limit.

After mauscript submission and BMC office confirmation that the manuscript is in the appropriate format (abstract, text, references, figures, tables, supplements), the manuscript is sent to the Boston office for initial review. Within 48 business hours, I assess the manuscript for its appropriateness for the *JCMR* readership and a determination as to its overall likely priority for publication. Approximately 5% of submitted manuscripts are deemed inappropriate for the *Journal* audience (non-CMR topic) or very unlikely to reach sufficient priority for acceptance (e.g., case reports/very small case series, unsolicited reviews). These manuscripts are returned to the author(s) within a week so as to expedite submission to a more appropriate journal. If appropriate, the authors are offered the opportunity to directly forward their manuscript to another BMC open-access publication.

For manuscripts deemed appropriate for consideration, an associate editor is assigned and reviewer assignments are then requested. Manuscript evaluations are simultaneously requested from up to 5 reviewers (with special consideration for the 2 author suggested reviewers) until confirmed acceptance has been received by 3 reviewers. Reviewers are asked to follow a specific format [1] and to return their review within 2 weeks of acceptance. We are fortunate to have nearly > 1000 registered reviewers (but are continuously interested in expanding our reviewer pool and encourage all members/innovators/leaders of the CMR field to apply to be a reviewer. If you are interested in becoming a *JCMR* reviewer, please contact our managing office: jcmroffice@scmr.org*.*

When at least two (of 3 agreed) reviews have been received by noon Friday, the manuscript is scheduled to be discussed at our associate editorial board meeting which is held every Tuesday from 9:30 to 10:30 a.m. ET. When I am out of town/unavailable, the associate editors continue to meet at that time so as to not delay the publication process. At each meeting, 4–12 manuscripts may be discussed. The manuscript decisions at that meeting include.

1. *Accept*
2. *Minor revision* No new experiments are requested, relatively minor text changes or analyses are requested; 30 day turn-around. These manuscripts are generally *not* returned to the reviewers for their assessment. We expect > 98% acceptance.
3. *Major revision* Substantial text and/or analyses are needed, a few additional experiments; 90 day turn-around. These manuscripts are sent back to the original reviewers to confirm that their concerns have been adequately addressed and ~ 60% acceptance is anticipated
4. *Denovo resubmission* Substantial new experiments/analyses are needed or change in manuscript focus; unlimited turn-around time. These manuscripts are sent back to the original reviewers to confirm their concerns have been adequately addressed and ~ 40% acceptance is anticipated.
5. *Decline* Authors are offered the opportunity to have their manuscript considered by another journal in the BMC family with inclusion of the *JCMR* reviews to expedite the process.

When a manuscript is accepted, I then edit the submission for *JCMR* style/abbreviations (see https://jcmr-online.biomedcentral.com/submission-guidelines/preparing-your-manuscript/abbreviations) before final submission to BMC for galley production. The galleys are first sent to the corresponding author and finally to me for final sign-off. I then identify a fingernail image for publication in *JCMR* and to accompany the @JournalofCMR twitter handle. The manuscript is usually published on-line within a week of my final sign-off.

Our target goal is than 60% of manuscripts will have a submission to first decision within 40 days of receipt, a process that is very dependent on timely reviews. If the two reviews markedly differ in their assessment/recommendation (~ 25% of the time) or the associate editor feels we need additional information, we may delay a decision until the third review has been received or solicit a fourth reviewer – a process that unfortunately can add a month or more to the review process. At our editorial meeting, we may also to seek the counsel of our statistical reviewer, Dr. Long Ngo. We try to alert the corresponding author if any of these situation occurs or the unusual occurance of our not being able to discuss all of the manuscripts on our weekly agenda (or the assigned associate editor is unable to participate).

We recognize that the process is not perfect. We may have not sent to the best reviewers (your suggestions help), or the best reviewers were unfortunately not available. Sometimes you will find the editorial decision is different from your perception of the review(s). This is because we do our best to objectively assess the science, presentation, and appropriateness for the *JCMR* audience. The review(s) help, but we also ask ourselves these four questions:

1. Is the study scientifically sound?
2. Are the Methods, Results, and Discussion appropriately presented?
3. Is the work novel? Does the study extend or clarify our current understanding or is it a confirmation of a prior report?
4. Will our readership be interested or informed by the topic?

Anonymized reviews are returned to the authors and are currently not available to our readers. We are currently working with BMC to be able to have *anonymized* reviews for published manuscripts available to you. I do not anticipate publication of submitted (but not accepted manuscripts) or inclusion of prior versions of an accepted manuscript with reviews as I am concerned this may be confusing to the reader.

## Conflict-of-interest, Reviews, SCMR Guideline/Position manuscripts and SCMR Committee papers

Conflict-of-interest manuscripts, those for which a member of the associate editorial board is either an author or closely associated with an author, are independently handled by a Guest Editor (Table 1) chosen by me. Neither I nor any of the associate editorial board are involved with reviewer selection or with manuscript decision. Our managing editorial office assists the Guest Editor with the administrative software/Editorial Manager. If a conflict-of-interest manuscript is accepted, the Guest Editor is recognized in the *JCMR* publication.

**Table 1:** 2019 *JCMR* Guest Editors

Mark Fogel
Matthias Friedrich
Robert Judd
Hildo Lamb
Debiao Li
Guy MacGowan
John Oshinski
Dana Peters
Martin Prince
Nathaniel Reichek
Michael Salerno
Matthias Stuber
Anne Marie Valente
Robert Weiss

The *JCMR* does not accept unsolicited reviews. Authors are encouraged to contact me before submitting any reviews. In general, reviews are authored by individuals considered experts in the field [2] and receive considerable attention/downloads. All solicited reviews follow the usual peer-review process. Several reviews are planned for 2021.

The *JCMR* is the official publication of the SCMR. As such, SCMR Guidelines and Position papers [3] endorsed by the Full (or Executive) SCMR Board(s) do *not* undergo peer review. I review these manuscripts for consistency with *JCMR* style and abbreviations. They are then published in an expeditious manner. We published several Covid-specific SCMR position papers this year [4–6].

In contrast to SCMR Guidelines and Position papers, SCMR Committee approved manuscripts undergo the usual *JCMR* peer review process albeit with an anticipation that they will ultimately be published in the *JCMR.*

## All Manuscripts Submitted to the Journal CANNOT be under Simultaneous Consideration by Another Journal

All work submitted to the *JCMR* must be original and not under consideration by another journal. While we encourage you to submit your work that may have been declined by another journal with the associated reviews and response to the reviewers, *manuscripts cannot be under simultaneous review by another journals.* This past year we had the very unusual situation where we became aware of a manuscript that was under simultaneous consideration by the *JCMR* and another cardiac imaging journal. After consultation and confirmation with the Editor-in-chief of the other journal, the manuscript was immediately withdrawn from further consideration and the corresponding author contacted.

## Reviewer Recognition—Gold Star Reviewers

**Fig. 4:**
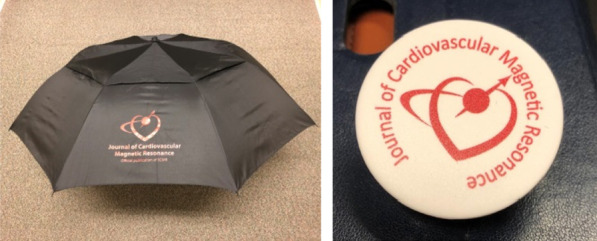
2019 JCMR Gold Star Reviewers and Guest Editors were offered their choice of an umbrella or phone holder pop-up; both embossed with the *JCMR* name and SCMR logo

Reviewers are a key component to the success of the *JCMR*. As a recognition of reviewers, at the 2020 SCMR Annual meeting in Orlando, Florida, USA we recognized our 124 “Gold Star” Reviewers for 2019 (Table 2). Gold Star reviewers are those individuals who reviewed at least 3 *JCMR* manuscripts in 2019, with reviews both of high quality and submitted on-time. In addition to public recognition at the meeting (Gold Star ribbon, *JCMR* booth listing, and intermission slide listing), each Gold Star Reviewer was offered a small gift (Fig. 4) as a token of our appreciation. Please join the ranks of *JCMR* reviewers and strive to be a Gold Star reviewer! As an added incentive, reviewers have the option to receive continuing medical education (CME) credit for providing a review!

**Table 2:** 2019 *JCMR* Gold Medal Reviewers

Anthony Aletras
Andrew Arai
Per Arvidsson
Ryan Avery
Adrianus Bakermans
W. Patricia Bandettini
Nicoleta Baxan
Rebecca Beroukhim
Ronald Beyers
Robert Biederman
Giovanni Biglino
Kenneth Bilchick
Konstantinos Bratis
Adrienne Campbell-Washburn
Andrea Cardona
Raymond Chan
YuCheng Chen
Byoung Wook Choi
Michael Chuang
Henry Chubb
Jeremy Collins
Christakis Constantinides
Francisco Contijoch
Erica Dall’Armellina
Rohan Dharmakumar
Chong Duan
Michael Elliott
Daniel Ennis
Emil Espe
Ahmed Fahmy
Li Feng
Juliano Fernandes
Pedro Ferreira
Paul Finn
Mark Fogel
Julio Garcia
Pankaj Garg
Nilesh Ghughre
Olaf Grebe
Lindsay Griffin
Lars Grosse-Wortmann
Ying Guo
Christopher Haggerty
Hassan Haji-Valizadeh
Ahmed Hamimi
Markus Henningsson
Lazaro Hernandez
Bobak Heydari
Peng Hu
Chenxi Hu
Nazia Husain
Adrian Ionescu
Tevfik Ismail
Ning Jin
Jason Johnson
Avinash Kali
Dinesh Kalra
Maria Kiaffas
Won Yong Kim
Gert Klug
Grigorios Korosoglou
Johannes Kowallick
Ramkumar Krishnamurty
Deborah Kwon
Raymond Kwong
Seung-Pyo Lee
Minjie Lu
Viviana Maestrini
Jeff Maki
Peirre-Yves Marie
Anthony Merlocco
Mehdi Moghari
Umberto Morbiducci
Av Naumova
Tomas Neilan
Thomas Neuberger
TD Nguyen
Declan O’Regan
Laura Olivieri
Eric Osborn
Jose Palomares
Farhad Pashakhanloo
Amit Patel
Ian Paterson
Eva Sophia Peper
Dana Peters
Stanislas Rapacchi
Shams Rashid
Kanishka Ratnayaka
Nathaniel Reichek
Toby Rogers
Idan Roifman
Sebastien Roujol
Tobias Rutz
Hajime Sakuma
Francesco Santini
Tobias Schaeffter
Andreas Schuster
Dipan Shah
Sujata Shanbhag
Sahar Swoleimanifard
David Sosnovik
Pascal Spincemaille
Monvadi Srichai-Parsia
Jordan Strom
Matthias Stuber
Peter Swoboda
Connie Tsao
Elizabeth Tunnicliffe
Martin Ugander
Ruud Van Heeswijk
Ralf Wassmuth
Gregory Wehner
Davide Wendell
John Whitaker
Timothy Wong
Yibin Xie
Hui Xue
Yang Yang
Alistair Young
Chun Yuan
Filip Zemrak
Chengcheng Zhu

## Continuing Medical Education (CME) *JCMR* Journal Club

In late 2017, we introduced on-line CME credit for the benefit of our clinician readers. This program has been a great success and now includes over 30 manuscripts members including 6 in 2019 [7–12] (Table 3). See http://scmr.peachnewmedia.com/store/provider/custompage.php?pageid=20 for the entire listing. In general, CME is offered for clinically oriented manuscripts. CME credit is provided at no cost for SCMR.

**Table 3:** 2019 JCMR CME manuscripts

Khan et al.	Association of left atrial volume index and all-cause mortality in patients referred for routine cardiovascular magnetic resonance: a multicenter study [7]
Gräni et al.	Comparison of myocardial fibrosis quantification methods by cardiovascular magnetic resonance imaging for risk stratification of patients with suspected myocarditis [8]
Freitas et al.	The amount of late gadolinium enhancement outperforms current guideline-recommended criteria in the identification of patients with hypertrophic cardiomyopathy at risk of sudden cardiac death [9]
Thompson et al.	Quantification of lung water in heart failure using cardiovascular magnetic resonance imaging [10]
Dabir et al.	Multiparametric cardiovascular magnetic resonance imaging in acute myocarditis: a comparison of different measurement approaches [11]
Holtackers et al.	Clinical value of dark-blood late gadolinium enhancement cardiovascular magnetic resonance without additional magnetization preparation [12]

**Fig. 5:**
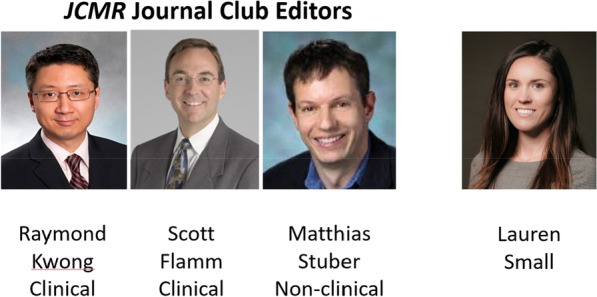
Inaugural *JCMR* Journal Club Editors – Drs. Raymond Kwong, Scott Flamm, and Matthias Stuber. Lauren Small from the SCMR management office has been instrumental in coordinating the administration of the monthly *JCMR* Journal Club series

A highlight of 2020 was the introduction and great success of our monthly one-hour webinar *JCMR* Journal Club held on the 2nd Wednesday of the month at 11am. The *JCMR* Journal Clubs are hosted by one of our 3 inaugural Journal Club Editors, (Fig. 5) Drs. Scott Flamm (clinical), Raymond Kwong (clinical) and Matthias Stuber (non-clinical). On a rotating basis, each editor choses a manuscript that was recently published in *JCMR*. After an author’s 25 min presentation, there is a spirited 30 min discussion. We currently offer CME for the chosen manuscript and hope to provide CME for Journal Club *attendance* in the coming year. Please join > 100 of your colleagues every month for an informative presentation and discussion! I very much have appreciated the strong administrativev assistance of Lauren Small (Fig. 5) from the SCMR managing office in coordinating the speaker presentations, Zoom operation and recording, and subsequent posting of the monthly *JCMR* Journal Club recording on the SCMR website.

## SCMR Case of the Week Series

While the *JCMR* does not accept case reports, for many years, the SCMR web site has an active “Case of the Week” (https://scmr.org/page/caseoftheweekLDGPG) series, currently coordinated by Dr. Sylvia Chen. In 2020, we will be publishing the 2019 Case series as a single manuscript. We plan to make this unified publication an annual occurrence in *JCMR* to allow for these illustrative cases to be more widely available to search engines.

## Editorial board

*JCMR e*ditorial board members are leaders in the CMR field and are expected to review up to 4 manuscripts/year. In 2020, we expanded the *JCMR* Senior Advisors group to now include Drs. Robert Edelman, Zahi Fayad, Victor Ferrari, Scott Flamm, Matthias Friedrich, Robert Judd, Stefan Neubauer, Roderic Pettigrew, Nathaniel Reichek, and Matthias Stuber. Many thanks to these leaders-in-the-field for lending us their expertise!

## Social media

I am very much a social media novice, but the *JCMR* continues to be very active on Twitter with the handle “JournalofCMR.” Tweets go out with the publication of each manuscript publication and announcing each Journal Club. This activity is coordinated by our two Social Media editors, Drs. Juan Lopez-Mattei and Purvi Parwani. According to Dr. Parwani, as of 12/10/2020, we had 3204 followers (a 32% increase over last year). For comparison, the *Journal of the American Society of Echocardiography* (*JASE*) has 2145 followers, the *Journal of Cardiac Computed Tomography* (*JCCT*) has 2483 followers, and the *Journal of Nuclear Cardiology* has 988 followers.

## Pohost and Pennell Awards

In recognition of the efforts of our inaugural editor-in-chief, Dr. Gerald M. Pohost, for the past 13 years, the *JCMR* has awarded the Pohost Prize to that manuscript deemed by the associate editors and editorial board to be the best/most important manuscript published in the prior year. The associate editors and I select the Pohost finalists (Table 4) and the entire editorial board votes on the top prize. At the 2020 SCMR annual meeting in Orlando, Florida, the 13^th^ Gerald M. Pohost Prize was awarded to Dr. Thompson for their manuscript” Quantification of lung water in heart failure using cardiovascular magnetic resonance imaging.” [10]. The Pohost Runner-up Prize was awarded to Dr. Nickander for “The relative contributions of myocardial perfusion, blood volume and extracellular volume to native T1 and native T2 at rest and during adenosine stress in normal physiology. [15].

**Table 4:** 2020 Gerald M. Pohost Award Finalists in alphabetical order by first author

Dabir et al.	Multiparametric cardiovascular magnetic resonance imaging in acute myocarditis: a comparison of different measurement approaches [11]
Femia et al.	Long term CMR follow up of patients with right ventricular abnormality and clinically suspected arrhythmogenic right ventricular cardiomyopathy (ARVC). [13]
Gotschy et al.	Characterizing cardiac involvement in amyloidosis using cardiovascular magnetic resonance diffusion tensor imaging [14]
Gräni et al.	Comparison of myocardial fibrosis quantification methods by cardiovascular magnetic resonance imaging for risk stratification of patients with suspected myocarditis [8]
Holtackers et al.	Clinical value of dark-blood late gadolinium enhancement cardiovascular magnetic resonance without additional magnetization preparation [12]
**Nickander et al**. **	**The relative contributions of myocardial perfusion, blood volume and extracellular volume to native T1 and native T2 at rest and during adenosine stress in normal physiology** [15]
Rodrigues et al.	Repaired coarctation of the aorta, persistent arterial hypertension and the selfish brain [16]
Seitz et al.	Impact of caffeine on myocardial perfusion reserve assessed by semiquantitative adenosine stress perfusion cardiovascular magnetic resonance. J Cardiovasc Magn Reson. 2019 Jun 24;21(1):33 [17]
Shusterman et al.	High-energy external defibrillation and transcutaneous pacing during MRI: feasibility and safety [18]
**Thompson et al**. *	**Quantification of lung water in heart failure using cardiovascular magnetic resonance imaging** [10]
Walheim et al.	Multipoint 5D flow cardiovascular magnetic resonance—accelerated cardiac- and respiratory-motion resolved mapping of mean and turbulent velocities [19]

^*^ 2020 Pohost Award Winner

^**^ 2020 Pohost Award Runner-up

At that meeting, we also presented the 2^nd^ Pennell Award in recognition of the foresight of our 2^nd^ Editor-in-Chief, Professor Dudley J. Pennell to transition the *JCMR* to the open-access platform. This decision (spearheaded by then SCMR Publications Committee chairman, Dr. Matthias Friedrich) markedly improved *JCMR*’s visibility and impact factor. The Pennell award is for that *original manuscript* that has most contributed to the *Journal’s* impact factor for the calendar year 3 years prior to the award. The 2^nd^ Dudley J. Pennell Prize was awarded to Dr. Captur for the publication, “A medical device-grade T1 and ECV phantom for global T1 mapping quality assurance-the T-1 Mapping and ECV Standardization in cardiovascular magnetic resonance (T1MES) program.” [20] with the runnerup prize awarded to Dr. Khan for “Top 100 cited articles in cardiovascular magnetic resonance: a bibliometric analysis” [21].

Stay tuned for the 14th Pohost and 4th Pennell Awards that will presented at the 23^nd^ Scientific Sessions of the *Society* this February!

## Survey Results for JCMR

The thoughts of our readership and suggestions for improvement are a constant source of reflection. This past summer, the SCMR and *JCMR* surveyed the membership with regards to their assessment of our *Journal*. We had nearly 170 respondants (80% clinicians or clinician-scientists) of which 75% reported reading the *JCMR* at least monthly and 80% felt that we that we had the right balance between clinical and technical publications. The most common access point was the SCMR website. Over 95% found value in the open-access format with 23% using *JCMR* as a source for CME and over 75% finding benefit of using the SCMR membership benefit for an 80% discount in the article publication fee (APC). 75% of respondents were highly likely or likely to submit their CMR research to *JCMR*, though competing imaging journals of *JACC: Cardiovascular Imaging* and *Circulation: Cardiovascular Imaging* were more appealing and the *European Heart Journal/Cardiovascular Imaging* was ranked similar to *JCMR* for clinical/translational manuscripts. The *Journal of Magnetic Resonance Imagin* was preferred for basic science/methods manuscripts with *JCMR* similar to *Magnetic Resonance in Medicine.* Suggestions for the future were for more reviews and a faster time to decision and these will be our focus in 2021.

## Manuscripts—WordPress

**Fig. 6:**
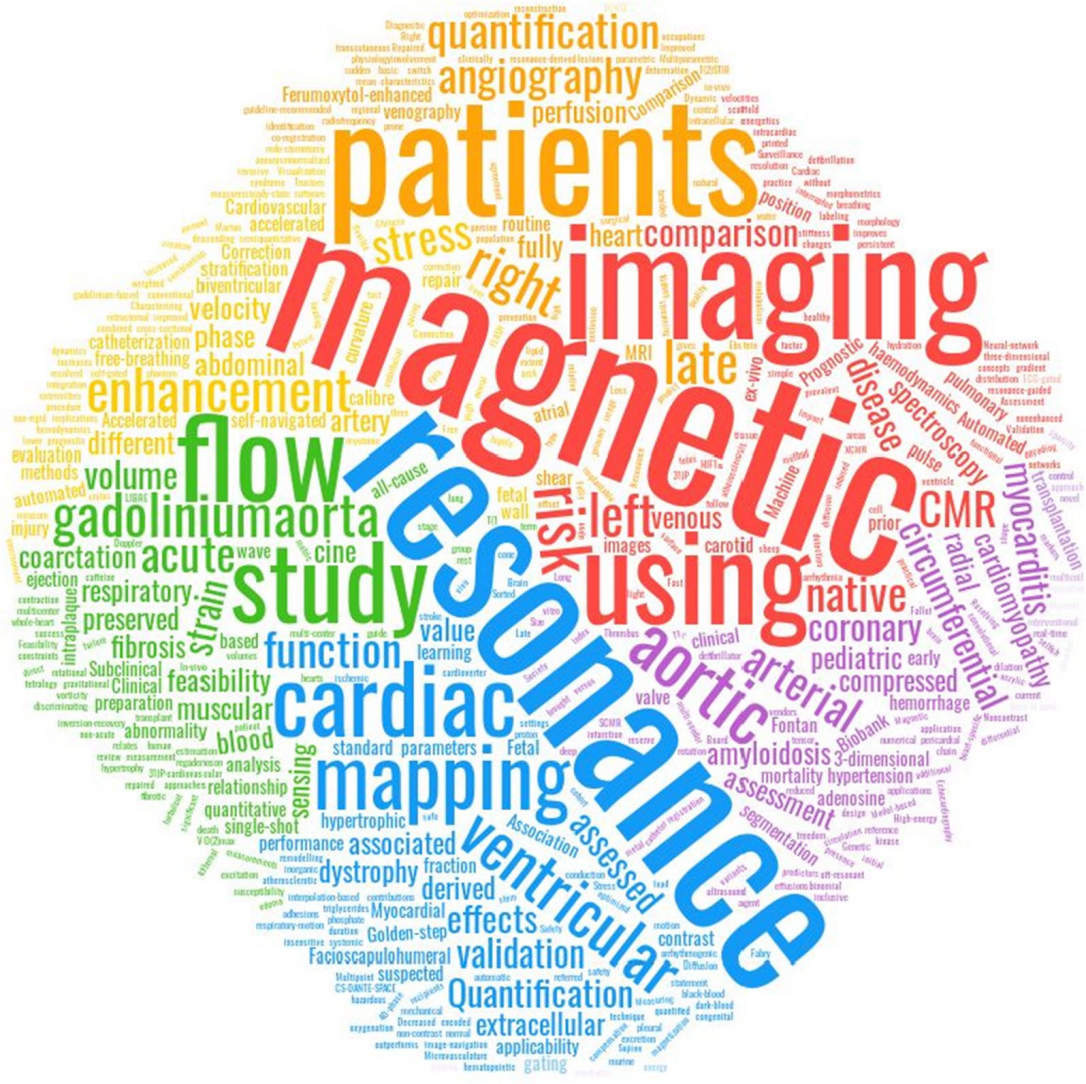
Wordplot derived from the titles of the 2019 JCMR publications

As I mentioned in the introduction, this year’s *JCMR* Annual Review is different from the format initiated by Dr. Dudley Pennell in 2010, as I am not including a thematic review of the prior 2 years of publication so as to minimize the resulting journal self-citation. As a global summary, methods, cardiomyopathy, vascular imaging, congenital heart disease and machine learning manuscripts predominated followed by flow, coronary artery disease, and population studies. To give you another overview perspective, I created a Wordplot of the 2019 titles (Fig. 6). The most common words were magnetic, cardiovascular, resonance, patients, imaging, myocardial, flow, cardiac, and mapping.

I hope you have found this “State of our *JCMR*” informative. I am the current captain, but as members of the *SCMR*, it is really your journal for which I thank you for allowing me to provide stewardship. I close by again thanking the entire *JCMR* team and you, our readership. We will try to get things better in 2021. I hope you that will continue to join us for the journey as we enter our 25^th^ year. Wishing you a happy, healthy, and safe 2021.

## Data Availability

Data sharing not applicable to this article as no datasets were generated or analyzed.

## Abbreviations

APC: Article processing charge
CME: Continuing medical education
*JCMR*: Journal of Cardiovascular Magnetic Resonance
SCMR: Society for Cardiovascular Magnetic Resonance
